# Orientation and contrast deviance examined: Contrast effects mimic deviant-related negativity yet neither produce the canonical neural correlate of prediction error

**DOI:** 10.1371/journal.pone.0299948

**Published:** 2024-03-15

**Authors:** Alie G. Male

**Affiliations:** Department of Psychiatry and Human Behavior, School of Medicine, University of California, Irvine, United States of America; Hong Kong Baptist University, HONG KONG

## Abstract

The visual mismatch negativity (vMMN) is a negative-going event-related potential (ERP) component that is largest somewhere between 100 and 300 ms after the onset of an unpredictable visual event (i.e., a deviant) in an otherwise predictable sequence of visual events (i.e., standards). Many have argued that the vMMN allows us to monitor our ever-changing visual environment for deviants critical to our survival. Recently, however, it has become unclear whether unpredicted changes in low-level features of visual input, like orientation, can evoke the vMMN. I address this by testing isolated orientation changes, to confirm recent findings, and isolated contrast changes, to determine whether other low-level features of visual input do not evoke the vMMN in a traditional oddball paradigm. Eighteen participants saw sequences of rare, unanticipated, and different deviant stimuli, interspersed among frequent, anticipated, and identical standard stimuli. Stimuli were Gabor patches. Neither deviant produced a vMMN. Therefore, changes in low-level visual properties of well-controlled stimuli–a stimulus in which one property can be manipulated while all others remain unaffected–like Gabor patches do not yield a vMMN.

## Introduction

Imagine one of our evolutionary ancestors moving through open forest on a windy day, looking for fruit. Its visual system and possibly attention are occupied primarily by the search for fruit of a certain shape and colour. Nevertheless, its visual system must remain attuned to the properties of the background, including the orientation of grasses and reeds, and the leaves, stems, and branches of trees that are constantly changing in the wind. Our ancestor needs to be able to detect alterations of those properties, such as a systematic change of the orientation of stems in one part of its visual field that might warn, for example, of the movements of a dangerous predator. It is likely that our ancestor’s brain had separate processes for finding food and for monitoring the visual scene for unanticipated changes. It is the latter the current study considers.

Predictive models of sensory processing are constructed from probabilities and priors that are learned from our surroundings and context moulded by our own worldly experience, they represent a core facet of the predictive coding theory, and are integral to how the brain processes changes in our immediate vicinity [1–3]. The first important feature of such models is that the brain uses them to predict future sensory input. Supposedly, the brain does this because that which is predictable requires little additional processing or processes like attention. The second feature is that the brain compares incoming input with that which the brain decides is predictable based on these models. If the incoming input is congruent with the predicted input, the brain maintains the model, but if the incoming input is incongruent with the predicted input, a prediction error occurs, and the brain updates the model so that it can better predict future sensory input.

Näätänen et al. [4] discovered the MMN; later interpreted as a neural correlate of prediction error. They found that, even though participants were not attending to the tones, event-related potentials (ERPs) from rare, different, unpredicted, *deviant*, tones produced a more negative voltage (i.e., negativity) than ERPs from a series of identical, *standard*, tones; this is the mismatch negativity (MMN) and it is typically shown in difference waves, calculated by subtracting the ERP to frequent standard tones from the ERP to infrequent deviant tones. The paradigm in which rare deviants appear interspersed among frequent standards is the oddball paradigm [5]. The visual analogue of the MMN is the visual MMN (i.e., vMMN) and occurs when a deviant visual input is detected and does not require attention to, or even consciousness of, deviance to occur.

Intuitively, this makes sense. Returning to our earlier example, our ancestor’s brain must have had a means for detecting unanticipated changes in the orientation of stems that could predict danger, even when concerned with foraging. The vMMN is thought to reflect this process. A frequently cited argument for prediction error is that it facilitates the constant need to adapt to one’s ever-changing environment [6–9], potentially signalling threat [10–16].

The vMMN is traditionally shown by subtracting the ERP for standards from the ERP for deviants, producing a deviant-minus-standard difference wave. The resulting negativity between 100 and 350 ms is a classic vMMN. Some have also described this negativity as deviant-related negativity (DRN), containing both deviant-related activity and adaptation-related activity [17]. A popular means of distinguishing adaptation-related activity from genuine deviant-related activity is the equiprobable control method [18]. In it, various stimuli with the same probability as the deviant replace the standards, thereby abolishing any probability effect specific to the deviant in the oddball paradigm. One can then compare ERPs to physically identical stimuli, except that one stimulus violates a regularity where the other does not. Accordingly, any deviant-minus-control difference wave should reflect pure deviance detection. Here, the resulting negativity between 100 and 350 ms is a genuine vMMN. With this control for adaptation, the number of studies showing no genuine vMMN to isolated orientation deviants is increasing [19, 20]. For the evolutionary reasons above, even changes in low-level properties of visual input—features that are normally processed at, or before reaching, the visual cortex (e.g., V1), such as orientation, contrast, phase, and spatial frequency—must be registered by the visual system, so we must reconcile the lack of vMMN to orientation deviants as resulting from some other reason.

One explanation is that some unanticipated changes in low-level features of visual input evoke the vMMN while others do not. Alternatively, irregularities in low-level features of visual input, when isolated, do not produce a genuine vMMN because their deviancy is resolved in a process that occurs before the vMMN, negating the need for a vMMN to these deviants [19]. I intend to confirm this with orientation deviants and test the assertion with contrast deviants. Stimulus luminance and contrast are arguably two of the most important sources of perceptual processing [21]. And, similar to the orientation changes that might predict danger to the foraging ancestor, contrast changes, particularly in the lower visual field, could signal an unanticipated shadow cast from above, potentially from a winged predator.

Recent work suggests that the difference between maximum and minimum luminance in a visual scene (i.e., luminance range) can moderate contrast sensitivity [22]. Moreover, stimulus brightness can affect neural activity associated with higher-order cognitive processes, such as processing valence-specific visual stimuli [23]. Contrast-dependent effects on popular visual illusions, such as the tilt illusion [24], have also been reported [25]. These findings emphasize the importance of cortical representations of visual contrast in early and late-stage processing.

There are only three studies investigating contrast deviance. In the first, Nyman et al. [26] found no vMMN to deviants with a lower Michelson contrast (M = .24) than the standard (M = .72). Instead, Nyman et al. [26] reported an enhanced positivity to higher contrast standards 200 ms after onset; this is a contrast effect. In the second study, Wei et al. [27] found a vMMN for deviants whose contrast was greater than that of the standard. However, Wei et al. [27] did not control for adaptation, thus limiting conclusions about the DRN. And, in a third study, there was no vMMN to irregular contrast increments within the multi-feature paradigm [19].

In the multi-feature paradigm [28], a feature, rather than the whole stimulus, is manipulated so that in a single sequence there can be several stimuli representing deviants for different features while simultaneously representing the standard for another so long as the feature is unchanged in at least two stimuli preceding a change in the same feature. A traditional standard in which no features are manipulated separates each deviant, or standard depending on the feature, and each deviant feature appears in a pseudo-randomized order. Although the paradigm is theoretically sound, relatively few have used the paradigm to show the vMMN (e.g., [8, 12, 15, 29, 30], so one cannot rule out the possibility that contrast deviants did not produce a vMMN due to having used the multi-feature paradigm instead of the oddball paradigm that was used in the first two studies.

To ensure that the reason there was no vMMN for contrast in Male et al. [19] was due to having used the multi-feature paradigm, the oddball paradigm is used in the present study. This will allow for confirming previous reports of no genuine vMMN to contrast deviants and, by extension, low-level features of visual input, and simultaneously confirm whether contrast deviants do produce a vMMN [27] or do not produce a vMMN [19, 26].

### The present study

The following study is designed to show whether isolated orientation deviants produce a vMMN, per recent works [19], and to delineate whether contrast deviants can produce a genuine vMMN that is distinct from a contrast effect. It compares orientation and contrast changes, after equating appreciable differences for each. However, there was no genuine vMMN for either visual feature. Bayesian analyses of deviant vs. control amplitudes provide some support for the null model.

## Method

### Ethics statement

The research was approved by the internal review board at Murdoch University (ethics permit 2015 208). Participants provided written informed consent prior to participation.

### Participants

According to G*Power v. 3.1.9.7. [31, 32], 14 participants were needed to replicate the genuine orientation vMMN reported by Kimura and Takeda [33] in their oddball condition at PO8 (–1.16 μV, *SD* = 1.59) to achieve a power of .80. This condition was chosen for the similarities in orientation deviance (details below) across studies. There is yet to be a study with a genuine vMMN for contrast decrements. Data for this experiment was collected between December 2015 and December 2016. Participants volunteered in return for course credit or the chance to win a $50 AUD voucher. Eighteen self-declared neurologically healthy adult participants (7 males, 17 right-handed) with normal or corrected-to-normal vision participated. Mean age was 32.7 (*SD* = 9.26) years with a range 18–48.

### Apparatus

Participants sat in a light-attenuated chamber facing a photometrically calibrated, 17-inch, colour, CRT monitor (Sony Trinitron Multiscan E230). The monitor showed 1280×1024 pixels at 100 Hz refresh rate. A PC running GNU Ubuntu (v16.04.4), Linux (v4.13.0), Octave (v4.0.0) [34], and Psychophysics Toolbox [35, 36] controlled the experiment and recorded behavioural responses. A chin rest stabilized participants’ heads 57 cm from the monitor. During the experiment, participants responded to targets by pressing a key on a 4-key response box with the index finger of their dominant hand. An iMac running NetStation 5.2 (EGI) recorded EEG data (S1–S6 Data).

### Stimuli

Stimuli were Gabor patches. No other visual stimulus affords control over all properties of the visual input such that one feature can be manipulated while all others remain unaffected. Gabor patches had a spatial frequency of 1.6 cycles per degree (cpd), a phase of one-quarter of a cycle (the centre of a white bar appeared in the centre of the screen), and a standard deviation of the Gaussian of 1° of visual angle. The visible part of the Gabor patch was approximately 4°. In half the blocks, stimuli appeared in the lower visual field. Here, the edge of the Gabor patch was .5° from the centre of the monitor. Due to reduced signal to noise ratio in these trials, analysis of the electrophysiology data was conducted separately and appears in the S2 File.

Visual contrast was calculated using Michelson contrast [37], based on its extensive use in the scientific community of vision research [38] as well as the studies outlined above. A pilot using categorical magnitude estimation [39, 40] ensured perceptual difference equivalence between orientation and contrast stimuli (S1 File). Categorical magnitude estimation is similar to magnitude estimation in most respects, except that the values assigned to stimulus intensity are limited as opposed to unrestricted as is the case in magnitude estimation [40]. Exponents and constants from the pilot data allowed for selecting values of orientation and contrast stimuli (details of the pilot in S1 File). There were four additional constraints in designing experimental stimuli. These were:

1. The difference between the standard and deviant orientation stimuli should be at least 33° because others have found a vMMN with orientation differences of 32.7° [33].
2. Cardinal orientations should be avoided to avoid confounds associated with the oblique effect; cardinal orientations tend to produce larger vMMNs than oblique orientations [41].
3. The deviant’s contrast should be less than the standard’s contrast to control for any adaptation-related differences—a lower contrast will not excite any unadapted neurons. This is also what Nyman et al. [26] did in their study.
4. The contrast of all orientation stimuli should be equal to that of the deviant contrast stimulus to avoid stark transitions between contrast and orientation blocks and facilitate potential comparisons between deviant stimuli across feature conditions.

**Fig 1:**
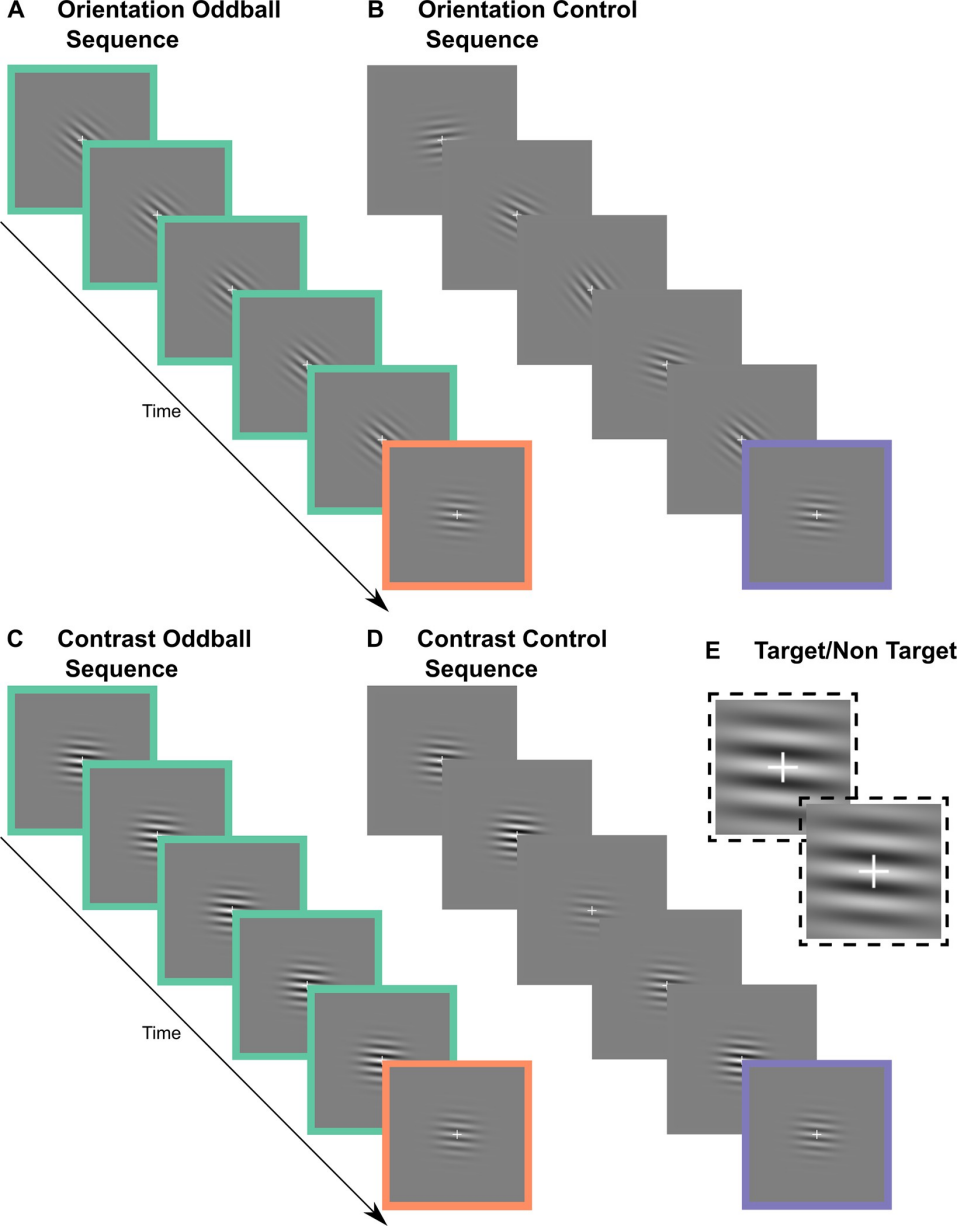
Illustrations of paradigm. Sequences in which stimuli are presented centrally. Deviant (orange) and control (purple) stimuli have the same probability (16.67%) of occurring in their respective sequences. At least three standard (green) stimuli separate deviants and each stimulus appears for 100 ms. **A. Orientation oddball sequence** in which the orientation of the deviant was 95° and the orientation of the standard was 128°. The standard and deviant values alternate across orientation blocks. **B. Orientation control sequence** in which the orientation of each stimulus is as follows: 84°… 117° … 139° … 106° … 128° … 95°… (… denotes the 400 ms ISI that is not shown). The Michelson contrast of all Gabor patches was .393 in all orientation blocks. **C. Contrast oddball sequence** in which the Michelson contrast of the deviant was always .393 and the Michelson contrast of the standard was always .846. **D. Contrast control sequence** in which the Michelson contrast of each stimulus is as follows: .544 … .997 … .695 … .242 … .846 … .363. The orientation of all Gabor patches within a contrast block was either 95° (as illustrated) or 128°. **E. Magnified fixation cross** during a target (bottom) and non-target (top) trial in a contrast block in which the Gabor patch Michelson contrast was .544 and the orientation was 95°.

To achieve a Michelson contrast difference that was equal to that of a 33° orientation difference in our contrast blocks, the contrast of standard stimuli was .846 and the contrast of deviant stimuli was .393. The remaining Michelson contrast values for the equiprobable control were .242, .544, .695, and .997. For orientation blocks, the Michelson contrast of the Gabor patch was always .393. The orientation of the standard and deviant was 128° and 95°, alternating in different blocks, chosen for their relative difference and position among the remaining orientation values: 84°, 106°, 117°, and 139° from vertical (0°), all of which were oblique angles. Fig 1 illustrates Gabor patches with these orientations (A−B) and contrast (C−D) values.

There was a white central fixation cross. The length of each bar of the fixation cross was .60° of visual angle; the width was .03° of visual angle. On target trials, the vertical bar of the cross grew in length (0.66° of visual angle). This is illustrated in Fig 1(E). Fixation-cross changes lasted 120 ms and onset was not synchronised with Gabor patch onset and offset.

### Procedure

Each block (*n* = 12) contained 480 trials and took 2.4 minutes to complete; each trial lasted for 500 ms and the inter-stimulus-interval (ISI) was 400 ms. Participants were free to take breaks between blocks.

There were four oddball blocks per feature each containing 80 deviant trials (17%). In half of the blocks, stimuli appeared centrally. The standard and deviant orientations reversed roles in half of the orientation oddball blocks; the standard and deviant contrasts did not because a deviant-minus-standard DRN for a contrast decrement could represent a genuine vMMN in a negative component. The number of standards separating deviants was randomised between 3 and 14. The average number of standards separating deviants was five. In equiprobable blocks (two per feature), all six values for a given feature appeared pseudo-randomly (i.e., repetitions not possible). Block order was randomized afresh for each participant. Fig 1 illustrates sequences for each deviant feature. Participants were asked to fixate on the always-present white fixation cross and respond to changes in it, ignoring all other stimuli. A response was correct when it occurred 150–1000 ms after target onset (i.e., change in fixation cross).

### EEG recording and analysis

Electroencephalogram (EEG) was recorded from 129 electrodes embedded within EGI’s dense-array HydroCel geodesic sensor net. Impedances were below the recommended and conservative 50 kΩ for dense-array recordings [42]. NetStation recorded EEG at a 500 Hz sampling rate. The signal was referenced to Cz during recording. EEG data were processed offline using the EEGLAB 14.1.1 [43] and ERPLAB 6.1.4 [44] in MATLAB 2022b (Mathworks Inc., USA). EEG activity was re-referenced to the common average. A low-pass 40 Hz Kaiser-windowed (beta 5.65) sinc finite impulse response (FIR) filter (order 184) was applied followed by a high-pass 0.1 Hz Kaiser-windowed (beta 5.65) sinc FIR filter (order 9056). Epochs were 500 ms long, featuring a 100 ms pre-stimulus time window. Standard trials immediately following the deviant were excluded. Epochs including amplitude changes exceeding 800 μV at any electrode were excluded from further analysis to remove artifacts that are not typical of eye movements or muscle artifacts.

A robust z-score for each electrode was calculated by replacing the mean by the median and the standard deviation by the robust standard deviation (0.7413 times the interquartile range) [45]. Electrodes with a z-score exceeding 3.0 were removed for interpolation later, provided at least four other channels surrounded them.

I computed independent component analysis (ICA) with AMICA [46] on the raw data (excluding noisy electrode) filtered with a 1 Hz high-pass (Kaiser-windowed sinc FIR filter, order 804, and beta 5.65) and 40 Hz low-pass filter [47]. Although segmented, the data was not baseline corrected. Winkler et al. [48] have shown that high-pass filters do improve ICA decompositions (improved reliability, independence, and dipolarity) and the de-mixing matrix can be easily applied to a linearly transformed dataset. Data was reduced to 32 components during the ICA computation and all 32 components were assessed for inclusion or exclusion in the final dataset.

I bipolarized data from electrodes above and below the right eye (electrodes 8 and 126) and outer canthi of both eyes (electrodes 1 and 32) to achieve vertical and horizontal EOG channels, respectively. Epochs containing amplitude changes exceeding ±60 μV at these EOG channels were identified for later rejection before applying the de-mixing matrix to the 0.1−40 Hz filtered data to ensure that trials in which participants moved their eyes or blinked were not included in the final dataset. All participants had at least 30 trials.

I assessed the ICA components using criteria described by Chaumon et al. [49] as well as ADJUST criteria [50]. Noise components were identified and removed based on low autocorrelation, low focal electrode or trial activity, high correlation with vertical or horizontal EOG, topography, or power spectrum. Thereafter, epochs previously marked for rejection were removed. Finally, data were interpolated for removed electrodes using spherical splines [51].

ERPs for the standard, deviant, and control stimuli were averaged before subtracting ERPs to controls and ERPs to standards from ERPs to deviants to produce two difference waves for each visual field and feature. Traditionally, the deviant-minus-standard difference wave reveals enhanced negativity due to a break from adaptation as well as prediction error (e.g., DRN), whereas the deviant-minus-control difference wave reveals prediction error only (e.g., vMMN). However, because the contrast deviant is always a contrast decrement and a lower contrast stimulus does not excite different neurons, DRN in the deviant-minus-standard difference wave could represent a vMMN for contrast deviants only.

For orientation stimuli, the mean number (*SD*) of epochs per participant (*N* = 18) in the grand average ERP was 484 (136) for standards, 121 (33) for deviants, and 123 (30) for controls. For contrast stimuli, the mean number (standard deviation) of epochs per participant in the grand average ERP was 487 (125) for standards, 121 (32) for deviants, and 66 (15) for controls. There were twice the number of controls for orientation compared to contrast stimuli because both versions of the orientation deviant appeared with equal probability among four other orientations in the equiprobable control blocks and both versions of the orientation deviant are included in the orientation control ERP.

I conducted temporal PCA on the individual standard, deviant, and control ERP data from all 129 electrodes for both features using the EP Toolkit 2.64 [52]. PCA was conducted for centrally presented stimuli separately (see S2 File for electrophysiology results for peripherally presented stimuli). I used a Promax orthogonal rotation (κ = 3) with a covariance relationship matrix and Kaiser weighting per Dien [53] and Dien et al. [54]. PCA reduces the data to components that account for most of the observed data. First the data was reduced to the number of components that explained more than 95% of the variance according to Horn’s parallel test [55]. Thereafter, components whose activity explained ≥0.02% variance in the data, or whose peak occurred after stimulus onset were kept, reducing the data from 13 to 10 principal components.

Using PCA, one can extract a single score per component and condition for statistical comparisons [56–58]. Each score represents the activity of a component (as if all other components that contribute to the EEG activity are absent), over all participants, in a single condition, at a specified electrode or ROI [59]. A vMMN component should appear as a negative deviant-minus-control difference score for orientation conditions or a negative deviant-minus-standard difference score for contrast conditions at the component’s minimum at PO electrode locations and would be largest between 100–300 ms [60] (for review, see Stefanics et al. [60]). The benefit of this approach is that it further reduces the effect of noise or activity unrelated to the component of interest.

A review of vMMN studies in which the manipulated property of visual input was a low-level feature shows the most used electrode or electrode clusters are at PO regions [19]. Three PO electrode clusters were defined. They are left parieto-occipital (L PO: E59, E60, E65, E66), midline parieto-occipital (M PO: E75, E72), right parieto-occipital (R PO: E91, E84, E85, E90). I extracted mean amplitudes from orientation ERPs between 197 and 207 ms at each PO ROI given this was the time window in which Kimura and Takeda [33] found the largest genuine vMMN in their oddball condition. For contrast, I extracted mean amplitudes between 150 and 200 ms at each PO ROI given this was the time window in which Wei et al. [27] reported the classic vMMN (no genuine vMMN reported).

I conducted Bayesian analyses of the data. For analysis of the behavioural data, I compared all models with the null model (*BF*_10_) and evaluated main effects and interactions by comparing the models containing a main effect or interaction to the equivalent models without the effect or interaction via the Inclusion Bayes Factor (*BF*_Incl_). For analysis of the mean amplitudes, I conducted Bayesian repeated-measures ANOVAs and Bayesian paired *t-*tests, using a medium prior (i.e., Cauchy prior whose width was set to 0.707). I report evidence in favour of the null where appropriate (*BF*_01_). I conducted Bayes Replication (*BF*_r0_) tests [61] for the contrast deviant-minus-standard conditions given a prior of: *t* = –7.167, at O2, for the classic vMMN (no genuine vMMN reported) in Wei et al. [27]. Bayes Replication (*BF*_r0_) tests provide evidence either favoring the idea that effects observed in the replication study match or exceed those in the original study, or supporting the hypothesis that no effect was discerned. To accomplish this, the posterior distribution derived from the original study serves as a knowledgeable prior, instead of the Cauchy prior distribution centered around 0 with a scaling factor of *r*  =  0.707 (the default “medium” effect size prior scaling).

In an exploratory analysis of deviance-related differences, I submitted microvolt-scaled component scores from the components with a peak latency between 50 and 350 ms to repeated-measures ANOVAs and two-tailed paired *t-*tests, using a medium prior (i.e., Cauchy prior whose width was set to 0.707).

## Results

### Behavioural

Table 1 shows hit rates and reaction times for correctly detecting increases in the size of the fixation cross for each condition for the 16 participants included in the central and lower periphery conditions.

**Table 1:** Mean (Standard deviation) hit rate (%) and reaction time (ms) for each block type and condition (N = 16).

Condition	Oddball	Equiprobable
*Hit Rate (%)*		
Central Presentation		
Orientation	67.5 (20.4)	72.1 (16.6)
Contrast	71.4 (16.8)	72.5 (15.5)
Lower Peripheral Presentation		
Orientation	80.6 (14.5)	82.2 (16.0)
Contrast	78.3 (14.3)	81.5 (17.4)
*Reaction Time (ms)*		
Central Presentation		
Orientation	526.4 (86.2)	519.9 (76.9)
Contrast	531.8 (76.8)	508.0 (73.0)
Lower Peripheral Presentation		
Orientation	479.4 (86.4)	471.5 (90.0)
Contrast	470.6 (83.6)	470.3 (89.6)

The model with the highest *BF*_10_ (the favoured model) was the model containing only the main effect of central vs. peripheral presentation for hit rate, *BF*_10_ = 489.680, and reaction time, *BF*_10_ = 40800.105, and there was strong evidence against including all other factors in explaining either hit rate or reaction time (all *BF*_Incl_ < 0.2). This is perhaps because stimuli appearing behind the fixation cross made fixation cross changes more difficult to see.

### Electrophysiology

**Fig 2:**
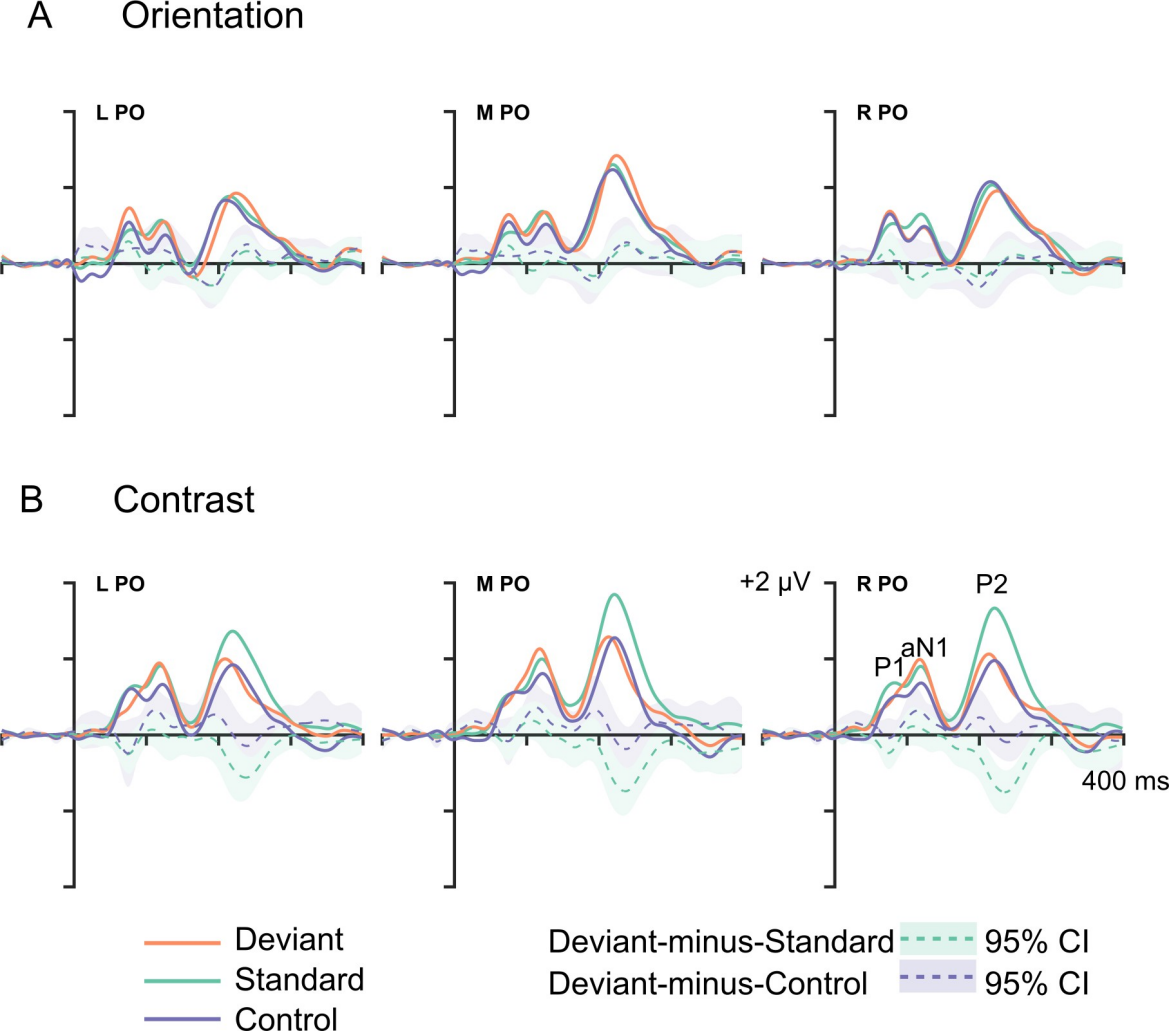
Grand average ERPs and difference waves for central orientation (A) and contrast (B) stimuli. ERPs for standard (green), deviant (orange), and control (purple) trials at the left (L), midline (M), and right (R) parieto-occipital (PO) regions. The lighter green and purple around the deviant-minus-standard (dashed purple) and deviant-minus-control (dashed green) difference wave, respectively, show the 95% confidence interval. Typical PO ERP components are shown at the R PO region of interest (ROI).

Fig 2 shows the ERPs for the standard (green), deviant (orange), control (purple) stimuli, deviant-minus-standard (dashed green) and deviant-minus-control (dashed purple) difference waves for orientation (A) and contrast (B) at PO regions. Difference waves include the 95% confidence intervals (CIs). Canonical ERP components appear at the right parieto-occipital (R PO) contrast condition. The P2 is the largest component, particularly for contrast standards due to their higher Michelson contrast compared to all other stimuli (S2 File for peripherally presented stimuli).

Table 2 shows no genuine deviance-related differences for either orientation or contrast. For deviant-minus-control conditions, the data are 1.211–3.344× and 5.895–8.109× more likely under the null model for orientation and contrast, respectively, according to directed Bayesian *t*-tests (*BF*_0-_).

**Table 2:** Paired Bayesian *t*-tests of the difference wave mean amplitudes (μV) at left (L), middle (M), and right (R) parieto-occipital (PO) regions for orientation and contrast conditions (df = 17).

	Deviant vs. Standard (Classic)				Deviant vs. Control (Genuine)			
	μV	*t*	*BF*_0-_	*BF*_01_	μV	*t*	*BF*_0-_	*BF*_01_
*Orientation*								
L PO	-0.124	-1.111	1.422	2.406	-0.100	-0.743	2.137	3.222
M PO	-0.027	-0.237	3.415	4.012	-0.036	-0.262	3.344	3.989
R PO	-0.108	-1.136	1.382	2.352	-0.178	-1.244	1.211	2.112
*Contrast*								
L PO	-0.053	-0.459	2.815	3.745	0.173	1.142	7.910	2.337
M PO	-0.112	-0.925	1.761	2.827	0.171	1.202	8.109	2.206
R PO	-0.168	-1.489	0.884	1.611	0.072	0.549	5.895	3.597

*Note*. *BF*_0-_ *=* Directed Bayes Factor value for likelihood data supports the null. *BF*_01_ = Two-tailed Bayes Factor value for likelihood data supports the null. Mean amplitudes from orientation ERPs are between 197 and 207 ms given this was the time window in which Kimura and Takeda [33] found the largest genuine vMMN in their oddball condition. Mean amplitudes from contrast ERPs are between 150 and 200 ms given this was the time window in which Wei et al. [27] found the largest classic vMMN (no genuine vMMN reported).

Given the large effect size reported in [27], replication tests did not provide support for the deviant-minus-standard difference at the L PO, *BF*_r0_ = 0.001, M PO, *BF*_r0_ = 0.004, R PO, *BF*_r0_ = 0.016. The data provide weak evidence in favour of the deviant-minus-standard difference at the R PO, *BF*_−0_ = 1.131 (L PO: *BF*_−0_ = 0.355, M PO: *BF*_−0_ = 0.568) with directed paired *t*-tests with default Cauchy prior.

**Fig 3:**
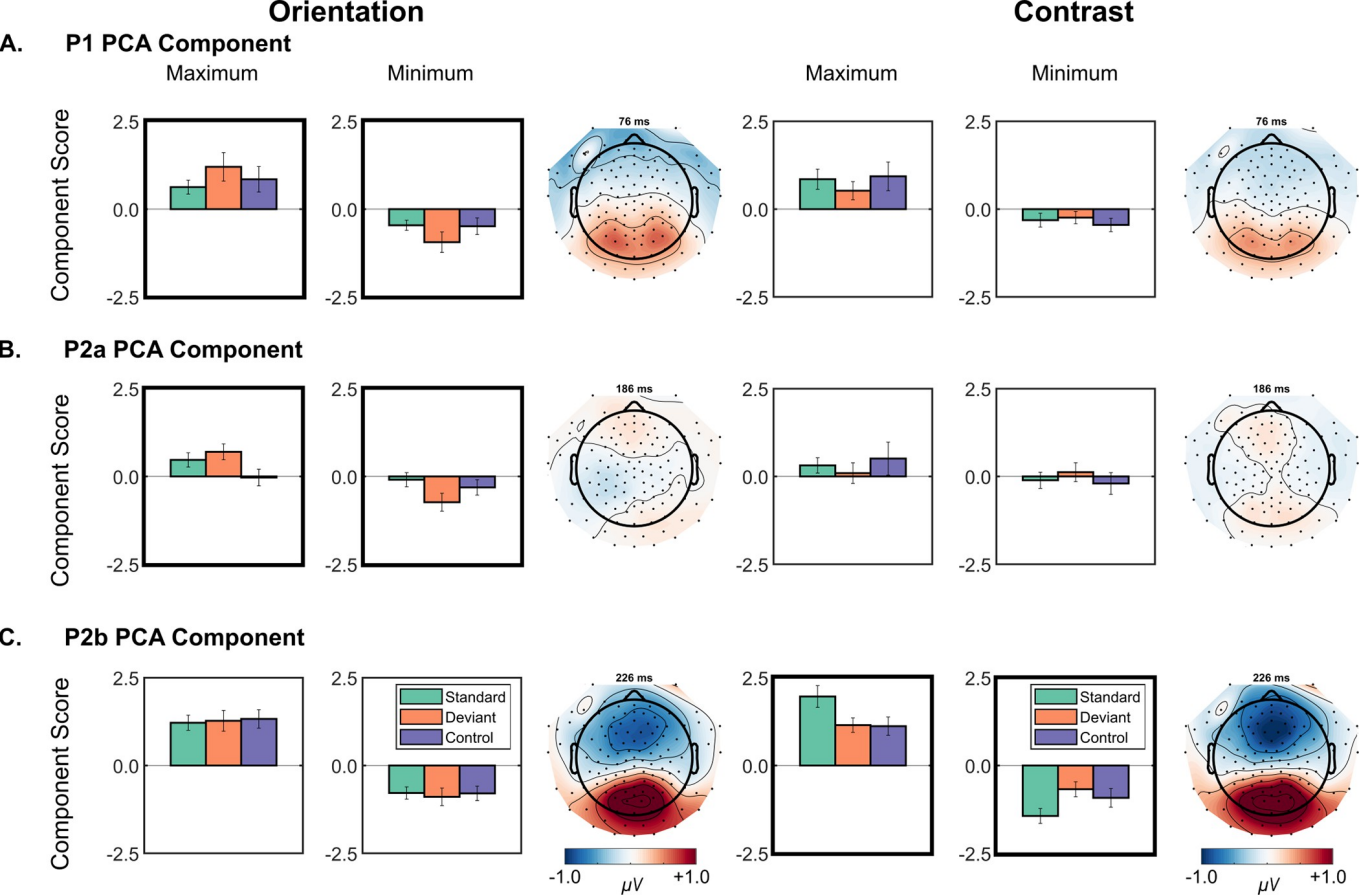
PCA results. Details of PCA components in which the data provide evidence in favour of the model containing stimulus type. **A. P1 PCA component results. B. P2a PCA component results. C. P2b PCA component results.** Topographical maps of each component show combined activity from standard, deviant, and control trials at peak latency. Component scores show means for standard (green), deviant (orange), and control (purple) stimuli at the site of peak positivity (Maximum) and negativity (Minimum). Error bars show ±1 standard error. Black boxes show conditions in which one or more paired Bayesian *t*-tests provide evidence in favor of differences between stimulus conditions.

Fig 3 shows the PCA components whose scores are more likely to distinguish between two or more stimulus types in either contrast or orientation conditions. These conditions are highlighted by black boxes in Fig 3. The topography and peak latency of these components are consistent with the P1, P2a, and P2b PCA components.

For the P1 PCA component, the data provide weak evidence in favour of the model with Stimulus Type for orientation stimuli at the site of maximal positivity, *BF*_10_ = 1.118, and site of maximal negativity, *BF*_10_ = 1.645. For the positive pole, Bayesian paired (two-tailed) *t*-tests provide weak evidence for the deviant vs. standard comparison only, *BF*_10_ = 1.340. For the negative pole, there is weak evidence for the deviant vs. control, *BF*_10_ = 2.025, as well as deviant vs. standard, *BF*_10_ = 1.012, comparisons only (remaining comparisons *BF*_10_ <1.0). Fig 3 helps to illustrate that this is the only component for which there is a deviant > standard *and* deviant > control difference indicative of genuine deviance as opposed to DRN (i.e., deviant vs. standard difference only).

For the P2a PCA component, the data provide moderate evidence in favour of the model with Stimulus Type for orientation stimuli at the site of maximal positivity, *BF*_10_ = 14.105, in which there is strong evidence for the deviant vs. control two-tailed comparison, *BF*_10_ = 7.329, but no evidence for the deviant vs. standard comparison, *BF*_10_ <1.0, and weak evidence for the standard vs. control comparison, *BF*_10_ = 1.975. At the site of maximal negativity, the data provide moderate evidence in favour of the model with Stimulus Type, *BF*_10_ = 6.559, owing to the deviant vs. standard comparison only, *BF*_10_ = 12.813 (remaining comparisons *BF*_10_ <1.0). The data does not support the model containing Stimulus Type (all *BF*_10_ <1.0) for the P1 and P2a PCA components scores for contrast stimuli.

Instead, the data provide very strong evidence in favour of the model with Stimulus Type for contrast stimuli for the P2b PCA component at the site of maximal positivity, *BF*_10_ = 2401.902, due to strong evidence for the standard vs. deviant, *BF*_10_ = 161.129, and standard vs. control comparisons, *BF*_10_ = 214.145 (deviant vs. control *BF*_10_ <1.0). Similarly, the data provide very strong evidence in favour of the model with Stimulus Type at the site of maximal positivity, *BF*_10_ = 78.554. Again, this reflects the standard vs. deviant, *BF*_10_ = 123.954, and standard vs. control comparisons, *BF*_10_ = 13.842 (deviant vs. control *BF*_10_ <1.0). This is the contrast effect.

## Discussion

The current study investigated whether orientation or contrast change evoked the vMMN; a correlate of prediction error. Although there was a negative-going difference that was characteristic of a vMMN in the deviant-minus-standard difference wave in the contrast condition (Fig 2), otherwise known as DRN, PCA confirmed that this reflects a larger P2 for higher contrast stimuli compared to lower contrast stimuli (Fig 3). Nyman et al. [26] reported a similarly enhanced positivity to higher contrast standards 200 ms after onset. Accordingly, the present study results corroborate Nyman et al. [26]‘s contrast effect and suggest that contrast deviants do not evoke the vMMN.

There were no deviant-minus-control differences in any component in the vMMN time-window. Outside of the vMMN time-window, there was some, albeit weak, evidence to suggest the data for the P1 are more likely to distinguish between deviants and controls in the orientation condition only. Comparable differences for well-controlled orientation changes have been reported by Male and O’Shea [62] and Male et al. [19]. In the present study, deviance-related differences (i.e., difference between deviant vs. standard–DRN–and deviant vs. control) appear at the site of peak negativity (i.e., negative pole) only. The effect is smaller (Cohen’s *d* = –0.403) than found previously (i.e., Cohen’s *d* = 3.378 [19] and Cohen’s *d* = 3.786 [62]. In prior experiments, the Michelson contrast of orientation stimuli was closer to .99 (Experiment 1 [62] and .60 (Experiment 2 [19]. Possibly, the reduced stimulus contrast in this experiment (M = .393) is responsible. The smaller P1s for orientation standards compared with contrast standards in Fig 2 helps to illustrate this contrast effect. Possibly, to show deviant-related differences, the stimulus-to-noise ratio must be high, and this is optimal when contrast is high.

The data did not indicate a likelihood for P1 PCA scores to differ between stimuli in the contrast conditions. Potentially, adaptation and deviance differences cancel each other out. Consider the following. If lower contrast stimuli produce smaller ERP amplitudes than higher contrast stimuli, as is seen in Fig 2 for the P2 (contrast effect = negative difference), but deviants produce larger ERP amplitudes than standard stimuli (deviance effect = positive difference e.g., [19, 62]), the sum of these effects may obscure both (negative difference + positive difference = no difference). Reversing the roles of deviant and standard contrast stimuli would help to test this although a control for higher contrast deviants would be necessary.

It is not likely that ISI or stimulus duration are responsible for failing to find the vMMN because they are identical to those others have used to test orientation deviance (e.g., [10, 63, 64]. The orientation difference is slightly larger than the orientation difference used by Kimura and Takeda [33]. Moreover, this is not the first to find that unexpected changes in low-level properties of visual input do not produce a genuine vMMN (e.g., for contrast [26], for orientation [19, 20, 62, 65]. So, there must be some other explanation for why some stimuli produce what appears to be a vMMN while others do not. Notably, Gabor patch stimuli are rarely used in vMMN research [19] and when they are used, they do not appear to evoke the vMMN when controlling for adaptation and attention (e.g., [19, 20, 62, 65]. In Male et al. [19], we compared conditions in which the stimulus were bars, like those used by Kimura et al. [63] or Gabor patches to determine whether the visual stimulus used was responsible for why some studies reported a genuine vMMN to orientation deviants (e.g., [63] whereas others did not (e.g., [20]). For each stimulus type, there was a condition in which there was a central fixation task (participants were asked to detect changes in a central fixation dot) vs. no fixation task (participants were asked to fixate and also detect changes in a peripheral element of the stimulus). Although there was some DRN in the deviant-minus-standard waveforms for the bar-edge condition, DRN was absent in the Gabor-fixation, and no condition produced a genuine vMMN.

Smout et al. [66] and Male and O’Shea [62] also found no DRN when using Gabor patch stimuli and fixation tasks. DRN may be more easily observed when using bar stimuli, as in [63]. File et al. [20] reported DRN when using peripherally presented bars, but not a genuine vMMN. Although the DRN reported by File et al. [20] may have reflected adaptation, it may also have been something else altogether. The electrophysiology and fixation data in Male et al. [19] provide some insight into what this may be. In the Kimura et al. [63] replication condition, fixation position was most variable due to the stimulus and task; the DRN was also largest. In conditions in which there was either a fixation task (bar-fixation) or controlled stimulus (Gabor-edge), the DRN was smaller. And when both a fixation task and controlled stimulus was used, the fixation position was most consistent and DRN was altogether absent. Accordingly, it may not be the stimulus itself that predicts DRN (e.g., bar vs. Gabor patch) but rather the extent to which the stimulus (or task) encourages inconsistent fixation position, thus affecting the input received by V1. Potentially fixation position is inadvertently contributing to the DRN in studies using non-Gabor stimuli and/or non-fixation tasks. Naturally, further vMMN studies employing both are prudent to confirm this. Still, the absence of a DRN in the orientation conditions in the current study is not seen as an indicator of a potential flaw in the design but rather a confirmation that other changing features of visual stimuli were not contributing to the results presented here.

The growing number of studies that show no vMMN following changes in low-level properties of well-controlled visual input like the Gabor patch [19, 20, 66–68] encourages continued investigation into how else these changes in visual input are resolved in the brain or, alternatively, closer inspection of the facets that yield a vMMN to these changes.

Undoubtedly, there is a means by which the visual system is processing these changes. If not, it would be unlikely that our foraging ancestor would have been able to detect those changes in visual properties, such as when a predator was approaching. A recent study by File et al. [69] found that change-blindness does not imply the absence of visual representations or deficits in distinguishing regular and irregular visual representations [69]. Their findings indicate that even for the most subtle perceptual changes, our brain’s encode such disparities. The question is, what neural signatures reveal detection? File et al. [69] reported posterior negativities ≥250 ms for undetected changes in scenery stimuli. This does not appear here. However, one would also anticipate that changes in simpler stimuli (e.g., Gabor patches) would produce a somewhat earlier neural signature based on: the stage in which low-level properties of visual stimuli (e.g., orientation, contrast) are processed in the visual system, prior evidence that undetected changes (i.e., change blindness) in the orientation of sinusoidal gratings are marked by distinct neural activity as early as 118–180 ms [70], and peak latency of vMMNs for similar, if not slightly more complex stimuli begin as early as (review of latencies in Male et al. [19]).

Potentially the early deviant-related positivity (EDRP) to isolated changes in low-level properties of visual stimuli reported elsewhere is involved. The current study indicates that this EDRP is readily observed in some conditions and not others (e.g., low stimulus intensity and peripheral presentation). Further investigation is vital to confirm neural signature of prediction error for isolated changes in low-level properties of visual input as well as the optimal conditions in which they are produced.

## Conclusions

The present study supports the conclusion that orientation and contrast deviants do not always evoke a genuine vMMN. This finding either suggests that for specific situations of visual change detection different processing mechanisms apply (i.e., mirrored in EDRP components other than the vMMN) or raises the question whether the vMMN depicts a generally reliable index of prediction error in the visual system.

## Supporting information

S1 File Categorical estimation pilot. (DOCX)

S2 File Electrophysiology results for peripherally presented stimuli. (DOCX)

S1 Table Bayesian analysis of principal components (PCs) of interest at the site of maximum positivity (Maximum) or negativity (Minimum). (DOCX)

S1 Data Log-transformed categorical estimation results. (XLSX)

S2 Data Hit rate and reaction time for centrally and peripherally presented stimuli in same participants. (XLSX)

S3 Data Electrophysiology data for centrally and peripherally presented stimuli. (XLSX)

S4 Data Difference wave mean amplitudes for centrally presented stimuli. (XLSX)

S5 Data Principal components for centrally presented stimuli. (XLSX)

S6 Data Principal components for peripherally presented stimuli. (XLSX)
