# Supplementary material for: Orientation and contrast deviance examined: Contrast effects mimic deviant-related negativity yet neither produce the canonical neural correlate of prediction error
Source: PLoS One. 2024 Mar 15;19(3):e0299948. doi: 10.1371/journal.pone.0299948 (PMC10942059; doi:10.1371/journal.pone.0299948)
Supplement: S1 File — (DOCX) [file pone.0299948.s001.docx]

**Supplementary Materials**

**S1. Pilot**

A categorical magnitude estimation pilot was used to ensure equal perceptual differences between orientation and contrast deviant and standard stimuli. Gabor patches had a spatial frequency of 1.6 cycles per degree of visual angle (cpd), a phase of one-quarter of a cycle, and a standard deviation of the Gaussian of 1° of visual angle. The visible part of the Gabor patch was approximately 4°. There was a white central fixation cross. The length of each bar of the fixation cross was .60° of visual angle; the width was .03° of visual angle.

For orientation blocks, the Michelson contrast of the Gabor patch was always .35. The orientation of the Gabor patch varied randomly and equally among 7°, 17°, 27°, 37°, 47°, 57°, 67°, 77°, and 87°. For contrast blocks, the orientation of the Gabor patch was always 47° clockwise from vertical (0°). Michelson's contrast of the Gabor patch varied randomly and equally among .1, .14, .2, .25, .35, .45, .6, .75, and .95. Stimuli appeared for 100 ms centrally or in the lower periphery in which the stimulus edge was .5° from the centre of the fixation cross.

At the beginning of each block, participants saw an anchor stimulus (also a Gabor patch) with which they were to compare each new stimulus. The Michelson contrast of the anchor was .35 for contrast blocks. The orientation of the anchor was 47° from vertical (0°) for orientation blocks. There were eight blocks per feature. Each block contained 54 trials, six per value of a feature. There were two naïve observers and AGM. Fig S1 shows the results.

**Fig S1. Mean magnitude estimates for orientation and contrast pairs.** Values in grey are logarithms. Values in parentheses are antilogarithms values. Antilogarithmic values for orientation are in degrees and antilogarithmic values for contrast are in Michelson. The black lines show the linear relationships from all three volunteers (red, blue, and green). Combined results from stimuli presented centrally and peripherally with their nearest edges 0.5° from fixation. Exponents are illustrated in each plot (rounded to two decimal places).

The difference in magnitude estimates between 1° and 34° (33° orientation difference) was 3.34 units. This difference was used to calculate equally different deviant and standard Michelson (M) contrast values for contrast stimuli. The deviant was .393 M. Due to a coding error; the standard was .846 M instead of .845 M. Given the size of the difference in contrast, the six contrast values were equally different from one another by one third of the deviant and standard difference (.151 M) in our equiprobable control. These were .242, .393, .544, .695, .846, and .997 M. Similarly, the orientation values for our equiprobable control were 84°, 95°, 106°, 117°, 128°, and 139° from vertical (0°).
