# Supplementary material for: Orientation and contrast deviance examined: Contrast effects mimic deviant-related negativity yet neither produce the canonical neural correlate of prediction error
Source: PLoS One. 2024 Mar 15;19(3):e0299948. doi: 10.1371/journal.pone.0299948 (PMC10942059; doi:10.1371/journal.pone.0299948)
Supplement: S2 File — (DOCX) [file pone.0299948.s002.docx]

**Supplementary Materials**

**S2. Electrophysiology Results for Peripherally Presented Stimuli**

Stimuli sequences appeared centrally or in the lower periphery. Several studies emphasize the benefit of lower periphery presentation in vMMN research [[70,71]](https://paperpile.com/c/ERIE6L/n3zz+BguE). Moreover, because cones in the peripheral part of the monkey retina respond 30 ms faster than cones in central vision [[72]](https://paperpile.com/c/ERIE6L/nE0h) and transient retinal ganglion cells, which have large receptive fields and preferentially respond to changes, disproportionately exist in the peripheral parts of the retina (e.g.,[[73]](https://paperpile.com/c/ERIE6L/orU0)), there may be greater deviant-related changes in the ERP waveforms for peripherally presented stimuli compared with centrally presented stimuli. Due to reduced SNR and no evidence of an effect of deviancy on ERPs and their components, analyses of peripherally presented stimuli were conducted separately.

During data pre-processing, two datasets were excluded for having fewer than 30 trials in one or more conditions, leaving 16 datasets (*N*=16). For orientation stimuli, the mean number (standard deviation) of epochs per participant in the grand average ERP was 477 (105) for standards, 120 (28) for deviants, and 121 (31) for controls. For contrast stimuli, the mean number (standard deviation) of epochs per participant in the grand average ERP was 508 (74) for standards, 125 (19) for deviants, and 63 (11) for controls.

Temporal PCA was used to reduce the data to the number of components that explained more than 95% of the variance according to Horn’s parallel test [[54]](https://paperpile.com/c/ERIE6L/CPtu). Thereafter, components whose activity explained more than 0.02% variance in the data, and whose peak occurred after stimulus onset were maintained, reducing the data from 15 to 8 principal components for peripherally presented stimuli. Despite this, there is still large variability in amplitudes in Fig S2, such that no CIs in the deviant-minus-standard or deviant-minus-control difference waves are below zero. Potentially reduced signal intensity of peripherally presented stimuli—due to having excited peripheral cells, not foveal cells—affected the overall signal to noise ratio in the visual cortices, obscuring deviance-related differences.

**Fig S2. Grand average ERPs and difference waves for peripherally presented orientation (A) and contrast (B) stimuli.** ERPs for standard (green), deviant (orange), and control (purple) trials at the left (L), midline (M), and right (R) parieto-occipital (PO) regions. The lighter green and purple around the deviant-minus-standard (dashed purple) and deviant-minus-control (dashed green) difference wave, respectively, show the 95% confidence interval.

Table S1 shows the results of exploratory analyses. Paired Bayesian *t*-tests were conducted for components showing a main effect of stimulus to determine if the effect was due to adaptation or deviance. The PCA component whose peak latency is consistent with the P2a shows the data provide evidence for a difference in both pairs including deviants. However, M1-M2 values show that this is in the opposite direction of deviant-related negativity (DRN). Instead, standard and control stimuli have larger (more positive) scores than the deviant. This is consistent with adaptation effect in contrast conditions only as the standard stimuli and average contrast in control condition have higher contrast stimuli than deviant stimuli.
