# Supplementary material for: Orientation and contrast deviance examined: Contrast effects mimic deviant-related negativity yet neither produce the canonical neural correlate of prediction error
Source: PLoS One. 2024 Mar 15;19(3):e0299948. doi: 10.1371/journal.pone.0299948 (PMC10942059; doi:10.1371/journal.pone.0299948)
Supplement: S1 Table — (DOCX) [file pone.0299948.s003.docx]

**Supporting Materials – Tables**

**Table S1**. *Bayesian Analysis of Principal Components (PCs) of Interest at the Site of Maximum Positivity (Maximum) or Negativity (Minimum).*

|  |  | **Maximum** | | | | | | **Minimum** | | | | | |
| --- | --- | --- | --- | --- | --- | --- | --- | --- | --- | --- | --- | --- | --- |
| **PC#** | **Time (ms)** | **Feature** | **ANOVA *BF*_10_** | **Stimulus1** | **Stimulus2** | **M1-M2** | ***t*-test *BF*_10_** | **Feature** | **ANOVA *BF*_10_** | **Stimulus1** | **Stimulus2** | **M1-M2** | ***t*-test *BF*_1_** |
| 2 | 144 | Orientation | 1.251 | Deviant | Standard | -0.131 | <1.0 | Orientation | <1.0 |  |  |  |  |
|  |  |  |  | Deviant | Control | -0.556 | 1.230 |  |  |  |  |  |  |
|  |  |  |  | Control | Standard | 0.425 | <1.0 |  |  |  |  |  |  |
|  |  | Contrast | <1.0 |  |  |  |  | Contrast | <1.0 |  |  |  |  |
| 4 | 210 | Orientation | 3.353 | Deviant | Standard | -0.449 | 8.166 | Orientation | <1.0 |  |  |  |  |
|  |  |  |  | Deviant | Control | 0.559 | <1.0 |  |  |  |  |  |  |
|  |  |  |  | Control | Standard | -1.008 | 2.778 |  |  |  |  |  |  |
|  |  | Contrast | <1.0 |  |  |  |  | Contrast | <1.0 |  |  |  |  |
| 5 | 92 | Orientation | 1.019 | Deviant | Standard | 0.024 | <1.0 | Orientation | <1.0 |  |  |  |  |
|  |  |  |  | Deviant | Control | -0.550 | <1.0 |  |  |  |  |  |  |
|  |  |  |  | Control | Standard | 0.574 | 1.057 |  |  |  |  |  |  |
|  |  | Contrast | <1.0 |  |  |  |  | Contrast | <1.0 |  |  |  |  |
| 7 | 180 | Orientation | <1.0 |  |  |  |  | Orientation | <1.0 |  |  |  |  |
|  |  | Contrast | 3.658 | Deviant | Standard | -0.346 | 3.635 | Contrast | <1.0 |  |  |  |  |
|  |  |  |  | Deviant | Control | -0.825 | 1.386 |  |  |  |  |  |  |
|  |  |  |  | Control | Standard | 0.479 | <1.0 |  |  |  |  |  |  |
| 8 | 256 | Orientation | <1.0 |  |  |  |  | Orientation | <1.0 |  |  |  |  |
|  |  | Contrast | 1.933 | Deviant | Standard | 0.318 | <1.0 | Contrast | 56.078 | Deviant | Standard | 0.012 | <1.0 |
|  |  |  |  | Deviant | Control | -0.544 | <1.0 |  |  | Deviant | Control | 1.239 | 6.494 |
|  |  |  |  | Control | Standard | 0.862 | 3.397 |  |  | Control | Standard | -1.227 | 40.645 |

*Note.* Paired Bayesian t-tests were conducted for PCs showing a main effect of stimulus to determine if the effect was due to adaptation or deviance. PC# 7 shows the data provide evidence for a difference in both pairs including deviants however this is in the opposite direction of deviant-related negativity (DRN). Instead, standard and control stimuli have larger (more positive) scores than the deviant. Where the data provide evidence for the alternative, the stimulus type with more positive (at maximum) or negative (minimum) score appears in a red or blue cell, respectively.
